# Immunohistochemical Expression of Integrin α_v_β_6_ in Surgically Resected Pulmonary Inflammatory Lesions Mimicking Malignancy on ^18^F-FDG PET/CT: Implications for the Specificity of ^68^Ga-Trivehexin PET/CT

**DOI:** 10.3390/biom16040602

**Published:** 2026-04-18

**Authors:** Muin Tuffaha, Amro Tuffaha, Wael Hananeh, Mohammad Khalifeh, Jenny Sonke, Michael Starke

**Affiliations:** 1 Institute of Pathology, Medical University Lausitz-Carl-Thiem, 03048 Cottbus, Germany; m.tuffaha@mul-ct.de (M.T.);; 2 Medical Clinic, Pulmonology, Medical University Lausitz-Carl-Thiem, 03048 Cottbus, Germany; 3 Faculty of Veterinary Medicine, Jordan University of Science and Technology, Irbid 22110, Jordan; 4 Faculty of Allied Medical Sciences, Jadara University, Irbid 21110, Jordan; 5 Department of Nuclear Medicine, Medical University Lausitz-Carl-Thiem, 03048 Cottbus, Germany

**Keywords:** integrin α_v_β_6_, PET/CT, ^68^Ga-Trivehexin, FDG, pulmonary lesions, inflammation, immunohistochemistry

## Abstract

**^18^**F-fluorodeoxyglucose (FDG) PET/CT is widely used for the evaluation of pulmonary lesions but lacks specificity, as increased FDG uptake is frequently observed in inflammatory and reparative processes. This limitation may lead to false-positive interpretations and unnecessary surgical resections. This study aimed to evaluate the immunohistochemical expression of integrin α_v_β_6_ in 18 surgically resected pulmonary lesions that were falsely classified as malignant on FDG PET/CT, in order to find out if ^68^Ga-Trivehexin PET/CT could have superior preoperative diagnostic specificity. Histopathological examination classified all lesions as non-neoplastic inflammatory processes of varying etiologies. Integrin α_v_β_6_ expression was detected in all immunohistochemically examined tissue specimens (18/18 cases (100%)), with moderate membranous overexpression in 2/18 cases (11.11%) and strong membranous overexpression in 16/18 cases (88.89%) observed in the alveolar and bronchial epithelium of inflammatory lung lesions. Our findings indicate that integrin α_v_β_6_ is upregulated not only in neoplastic lung tissue but also in inflammatory lesions, suggesting that integrin α_v_β_6_ may have limited specificity for distinguishing primary neoplastic from inflammatory pulmonary lesions when used alone. Its interpretation requires integration with other clinical imaging modalities and histopathological data.

## 1. Introduction

The preoperative differentiation of malignant and benign pulmonary lesions remains a critical diagnostic challenge. Conventional metabolic imaging with ^18^F-fluorodeoxyglucose (FDG) PET/CT has transformed oncologic staging but is limited by non-specific uptake in inflammatory and reparative processes, frequently leading to false-positive interpretations and unnecessary surgical interventions. In our tertiary care center, over the past three years, 18 patients underwent surgical treatment, including wedge resection, segmentectomy or lobectomy for lung masses initially classified as malignant based on high FDG uptake, yet subsequent histopathologic analysis revealed exclusively non-neoplastic inflammatory lesions of various etiologies. This highlights an urgent need for more specific molecular imaging biomarkers to reduce avoidable morbidity from unwarranted surgery.

Emerging evidence suggests that PET radiotracers targeting integrin α_v_β_6_, rather than glucose metabolism alone, may improve the characterization of pulmonary lesions [1,2]. Integrins are a family of transmembrane heterodimeric cell adhesion receptors composed of α and β subunits [3]. Integrin α_v_β_6_ is minimally expressed in normal adult epithelial cells, but it is rapidly upregulated in proliferating epithelial cells during injury, repair, and remodeling [4,5]. In addition to its selective upregulation on neoplastic epithelial cells, it has gained interest as a molecular imaging target in oncology because it plays a key role in activating latent transforming growth factor-β (TGF-β), thereby linking epithelial responses to inflammation, fibrosis, and tumor progression [6,7]. Radiotracers targeting integrin α_v_β_6_, such as ^68^Ga-Trivehexin, have shown favorable specificity for different carcinoma types and high image contrast in early clinical investigations, correlating PET uptake with immunohistochemical integrin expression. Preliminary data suggest integrin-targeted PET may distinguish malignant from inflammatory or fibrotic processes with greater specificity than metabolic imaging alone [8,9,10].

^68^Ga-Trivehexin PET/CT, an integrin α_v_β_6_-targeted PET imaging modality, has demonstrated promise across multiple malignancies; however, its diagnostic performance in distinguishing malignant lung neoplasms from inflammatory mimics has not yet been systematically evaluated. Specifically, there is a paucity of histopathological correlation studies evaluating whether immunohistochemical expression patterns of integrin α_v_β_6_ in benign inflammatory pulmonary lesions correspond to the differential uptake observed with ^68^Ga-Trivehexin PET imaging. Such molecular phenotype profiling may identify epithelial signatures capable of refining preoperative imaging algorithms and potentially reducing unnecessary surgical resections in lesions misclassified by ^18^F-FDG PET [11].

Therefore, the current retrospective immunohistochemical study analyzes integrin α_v_β_6_ expression in 18 surgically resected lung lesions initially misinterpreted as malignant on FDG PET/CT but later confirmed as inflammatory. The current research aims to evaluate whether the expression of integrin α_v_β_6_ in non-neoplastic reactive epithelial cells could limit the specificity of integrin α_v_β_6_-targeted imaging with ^68^Ga-Trivehexin PET/CT for preoperative discrimination of benign from malignant pulmonary pathology and reduce unnecessary lung resections.

## 2. Materials and Methods

A total of 18 patients with radiologically suspicious lung masses, examined between October 2022 and September 2025 and with tumor-negative primary transbronchial lung biopsy results, underwent ^18^F-FDG PET/CT for further therapy management and staging. Those lesions demonstrated high FDG uptake, with elevated SUVmax, SUVpeak, MTV, and TLG relative to the surrounding lung parenchyma and mediastinal blood pool, and were accordingly classified as highly suspicious for malignancy. All 18 patients underwent surgical treatment by wedge resection, segmental lung resection, or lobectomy. On gross examination of the submitted formalin-fixed surgical specimens, all macroscopically suspicious lesions were entirely embedded in paraffin blocks and processed routinely according to a standard protocol [12] for subsequent histopathological and immunohistochemical evaluations.

The ^18^F-FDG PET/CT institutional protocol was conducted according to the standard international guidelines [13].

Following confirmation of the histopathological diagnosis, immunohistochemical analysis was performed on the diagnosed inflammatory lesions to detect integrin expression according to a previously published study [14].

Integrin α_v_β_6_ expression on stained slides was assessed at the epithelial cell membrane. Staining intensity was evaluated semi-quantitatively using a four-point scale (negative, weak, moderate, or strong) [15], considering the proliferation and distribution patterns of alveolar and bronchial epithelial cells within the inflammatory lesions. Scoring was performed independently by a certified pathologist.

Different imaging and histopathological figures were taken as representative examples selected to illustrate the typical imaging–pathology correlation found across the current study, rather than exhaustive case documentation.

This retrospective study was conducted in accordance with the ethical standards of the institutional and national research committees and with the principles of the Declaration of Helsinki. The study protocol was reviewed and approved by the ethics committee of the State Medical Association of Brandenburg (approval number: 2026-37-BO-ff; approval date: 14 January 2026).

## 3. Results

The preoperative **^18^**F-FDG PET/CT metrics, including SUVmax, SUVpeak, MTV and TLG values, alongside the specific histopathological findings and integrin α_v_β_6_ expression patterns for all examined cases, are summarized in Table 1. Histopathological analysis of all 18 lung specimens revealed no evidence of malignancy; instead, inflammatory, fibrotic, and granulomatous lesions of various etiologies were identified (Table 1). All 18 radiologically suspicious pulmonary lesions, initially interpreted as malignant based on high 18F-FDG uptake, were ultimately diagnosed as inflammatory lesions of varying etiologies based on histopathological examination. Histopathological examination of the inflammatory lesions demonstrated a consistent pattern across all analyzed samples, characterized by marked proliferation of alveolar and bronchial epithelial cells surrounding areas of necrosis or active inflammation. These epithelial cells also showed strong integrin α_v_β_6_ expression, forming a three-dimensional circumferential zone of dense integrin α_v_β_6_ positivity. This configuration confers high affinity for ^68^Ga-Trivehexin and provides a plausible explanation for the observed ^68^Ga-Trivehexin PET/CT positivity. Accordingly, the increased tracer uptake initially interpreted on PET/CT as suggestive of malignancy represented false-positive findings, resulting in clinical misinterpretation prior to histopathological confirmation.

**Table 1:** Clinicopathological characteristics, FDG PET/CT metabolic parameters, and integrin α_v_β_6_ expression in inflammatory pulmonary lesions (*n* = 18).

Case #	Surgical Procedure	SUVmax^a^	SUVpeak^b^	MTV^c^	TLG^d^	Histopathological Diagnosis	Integrin α_v_β_6_ Expression Score
1	Segmentectomy	9.36	2.96	0.28	1.54	Organizing pneumonia	Strong (Figure 1)
2	Segmentectomy	1.41	0.68	0.72	0.58	Organizing pneumonia with abscess formation	Strong
3	Wedge resection	4.55	1.10	0.14	0.38	Organizing pneumonia	Strong
4	Segmentectomy	3.29	1.89	1.92	3.36	Necrotizing granulomatous inflammation	Strong (Figure 2)
5	Lobectomy	15.06	8.99	4.86	37.84	Necrotizing granulomatous inflammation	Strong
6	Segmentectomy	16.60	10.32	5.04	45.93	Non-necrotizing granulomatous inflammation/sarcoidosis	Strong
7	Lobectomy	10.01	6.97	3.89	20.95	Abscess-forming pneumonia due to actinomycosis	Strong
8	Wedge resection	3.05	1.96	10.15	16.26	Necrotizing granulomatous inflammation	Strong
9	Wedge resection	9.33	2.74	0.24	1.37	Necrotizing granulomatous inflammation with fibrinoid necrosis/rheumatoid nodule	Strong (Figure 3)
10	Wedge resection	5.07	2.70	0.91	2.68	Abscess-forming aspiration pneumonia due to actinomycosis	Strong (Figure 4)
11	Wedge resection	9.18	3.10	0.30	1.72	Chronic necrotizing pulmonary aspergillosis	Strong (Figure 5)
12	Lobectomy	3.36	2.72	3.29	7.29	Organizing pneumonia	Moderate
13	Segmentectomy	13.13	8.15	10.34	75.72	Necrotizing granulomatous and abscess-forming inflammation	Strong
14	Segmentectomy	5.53	2.54	1.36	4.31	Pulmonary aspergilloma	Moderate
15	Wedge resection	18.07	9.90	2.70	27.36	Granulomatosis with polyangiitis	Strong (Figure 6)
16	Wedge resection	4.71	1.33	0.20	0.55	Necrotizing granulomatous inflammation	Strong (Figure 7)
17	Wedge resection	NA^e^	NA	NA	NA	Organizing pneumonia	Strong
18	Wedge resection	NA	NA	NA	NA	Organizing pneumonia	Strong

a. SUVmax: the highest single-voxel SUV (maximum metabolic activity). b. SUVpeak: the average SUV within a small fixed volume (1 cm^3^). c. MTV: Metabolic Tumor Volume. d. Total Lesion Glycolysis: overall metabolic activity. e. NA: data not available.

**Figure 1:**
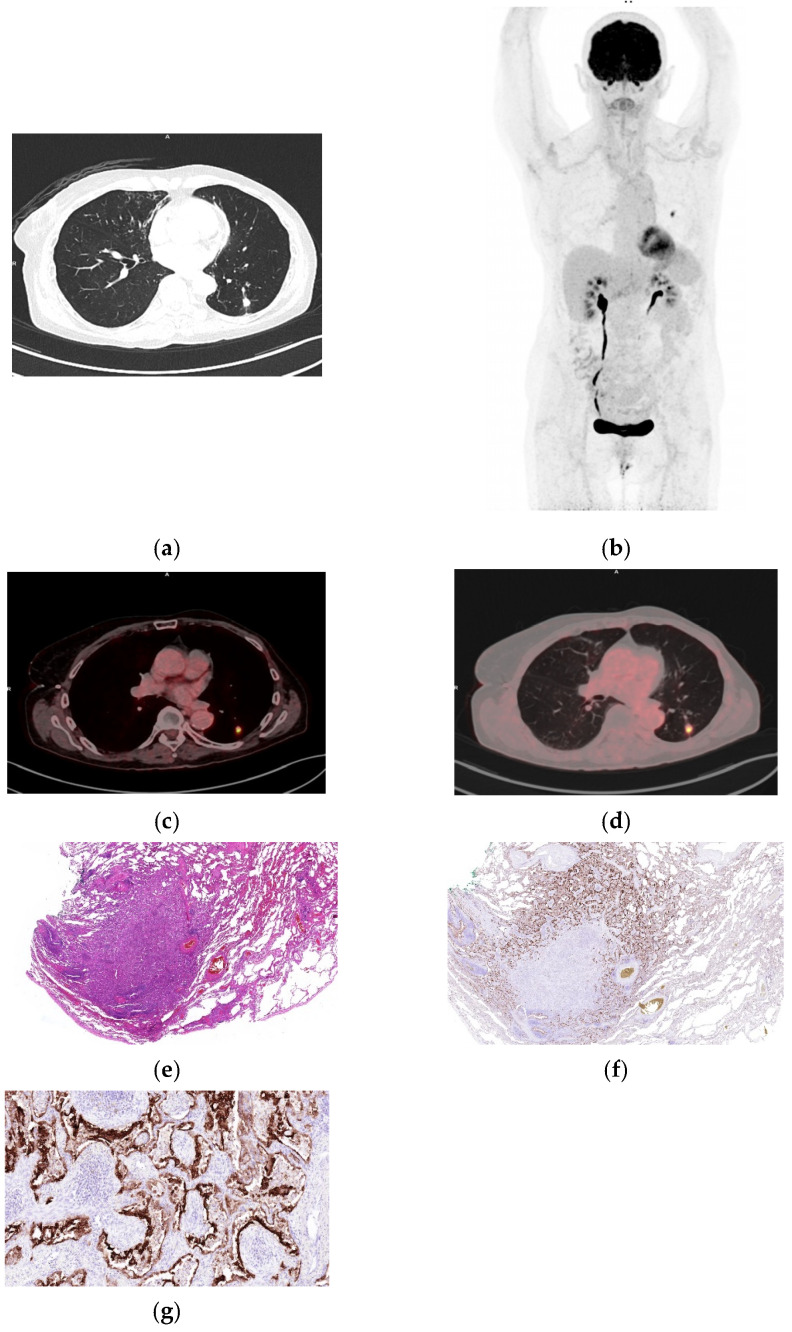
Representative case of organizing pneumonia mimicking malignancy on **^18^**FDG PET/CT. (**a**) Axial CT image demonstrating a well-defined lesion in the left lower lobe. (**b**) Whole-body maximum intensity projection (MIP) showing focal FDG uptake. (**c**,**d**) Fused axial ^18^FDG PET/CT images (soft tissue and lung windows) demonstrating increased tracer uptake (SUVmax 9.36 and SUVpeak 2.96). (**e**) Organizing pneumonia, lesion sharply demarcated from the surrounding lung parenchyma, associated with fibrosis and a chronic inflammatory cell infiltrate (H&E, 40×). (**f**) Immunohistochemical stain demonstrating membranous positivity of integrin α_v_β_6_ in alveolar and bronchial epithelium (40×). (**g**) Higher-power view demonstrates strong integrin α_v_β_6_ expression in proliferating alveolar and bronchial epithelium surrounding fibrotic alveoli (200×).

**Figure 2:**
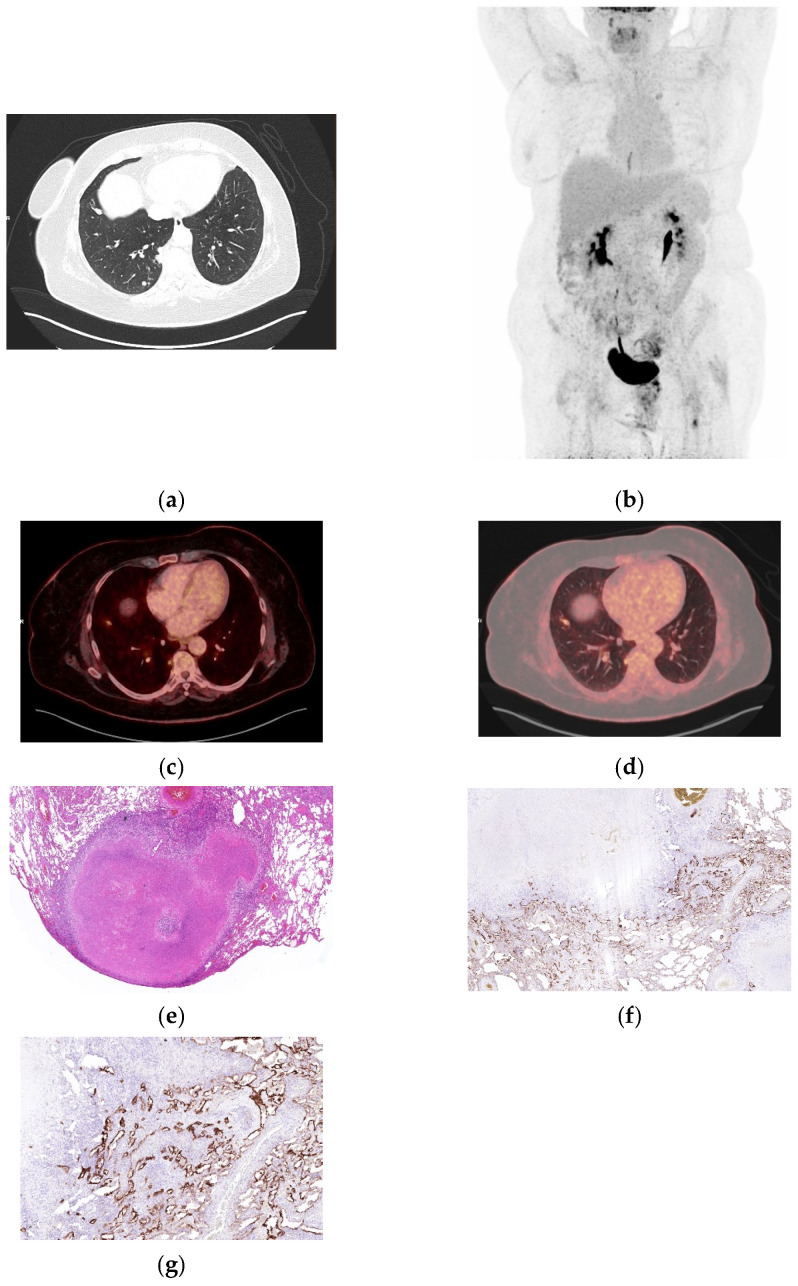
Necrotizing granulomatous inflammation with moderate FDG uptake. (**a**) CT demonstrating multiple pulmonary nodules in the left upper and right lower lobes. (**b**) Whole-body MIP demonstrating mild tracer uptake. (**c**,**d**) Fused axial **^18^**FFDG PET/CT (soft tissue window and lung window) with nodular uptake (SUVmax 3.29 and SUVpeak 1.89). (**e**) Circumscribed extensive subpleural parenchymal necrosis with surrounding lymphohistiocytic granulomatous inflammation (H&E, 40×). (**f**) Immunohistochemistry for integrin α_v_β_6_ shows positive alveolar and bronchial epithelium encircling the necrotic area (40×). (**g**) At higher magnification, there is marked proliferation of alveolar and bronchial epithelium with strong integrin α_v_β_6_ immunoreactivity adjacent to the necrotic area (200×).

**Figure 3:**
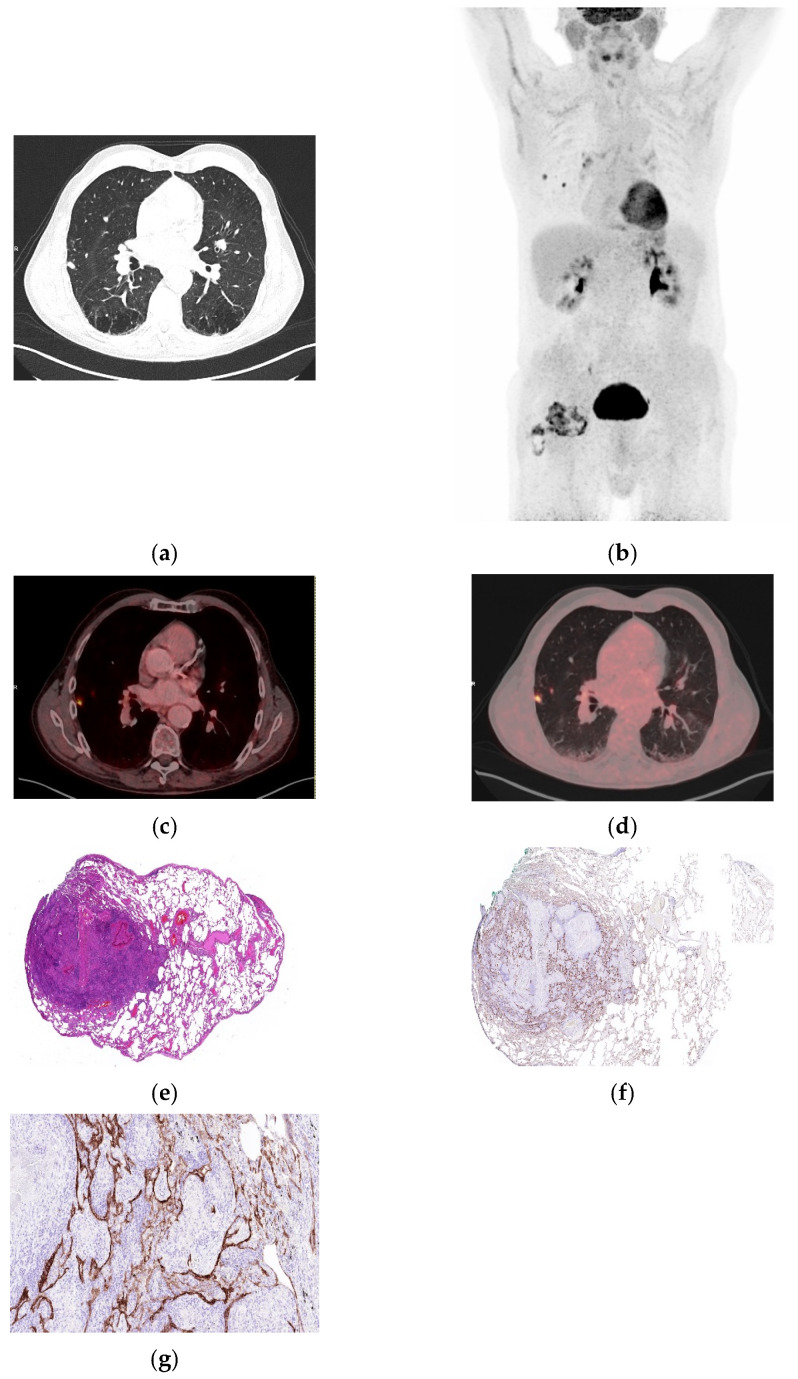
Necrotizing granulomatous inflammation consistent with rheumatoid nodule and high FDG uptake. (**a**) Axial CT demonstrated bifocal pulmonary nodules in the right upper and middle lobes, associated with mildly hypermetabolic bihilar and mediastinal lymphadenopathy. (**b**) Whole-body MIP showing increased tracer uptake. (**c**,**d**) Fused axial ^18^FDG PET/CT images (soft tissue and lung windows) demonstrating hypermetabolic nodules (SUVmax 9.33 and SUVpeak 2.74). (**e**) Lung wedge resection showing necrotizing granulomatous inflammation with fibrinoid necrosis consistent with rheumatoid nodule (H&E, 40×). (**f**) Immunohistochemical staining for integrin α_v_β_6_ demonstrates a membranous staining in the alveolar and bronchial epithelium (40×). (**g**) Higher magnification, exhibiting marked proliferation of alveolar and bronchial epithelium with strong integrin α_v_β_6_ expression (200×).

**Figure 4:**
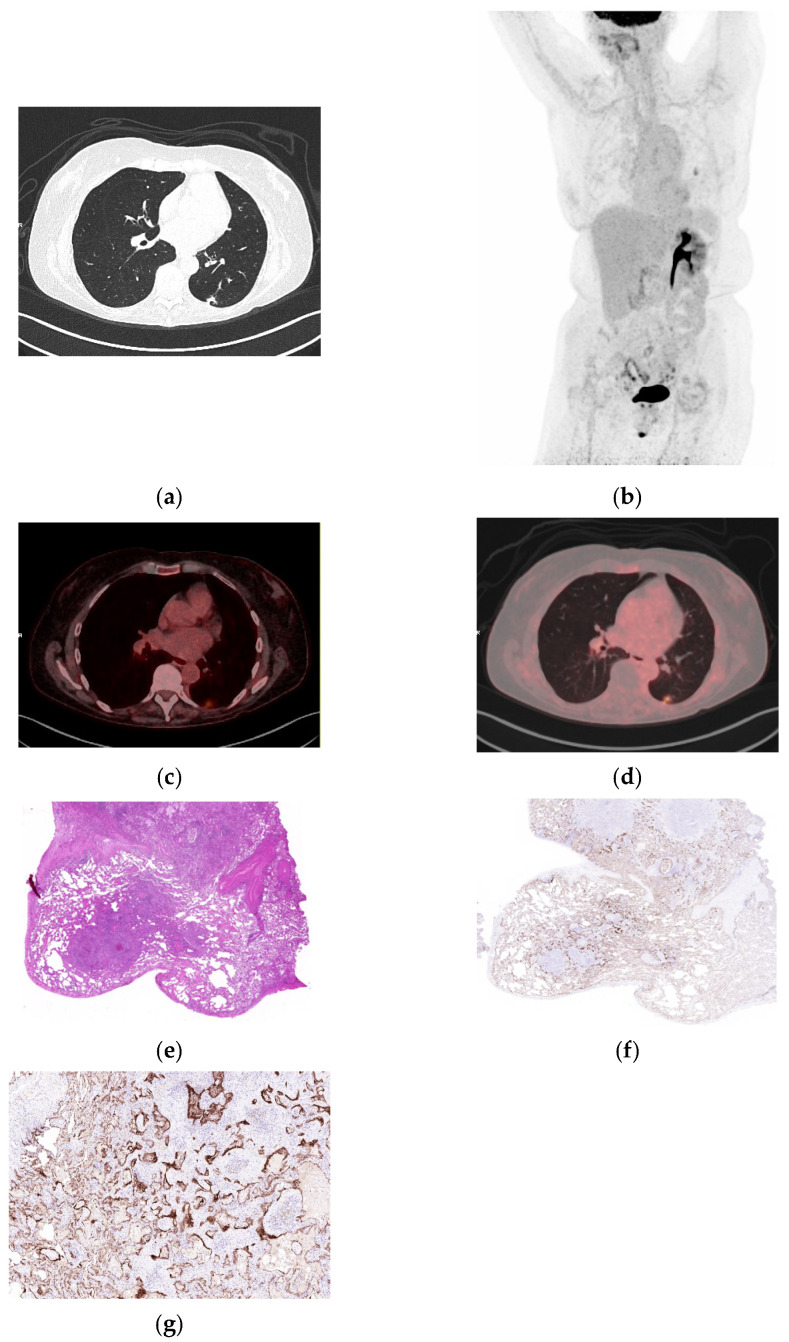
Aspiration pneumonia with abscess formation mimicking malignancy on ^18^FDG PET/C. (**a**) Axial CT shows a new single subpleural lesion in the left upper lobe. (**b**) Whole-body MIP showing focal FDG uptake. (**c**,**d**) Fused axial ^18^FDG PET/CT images (soft tissue and lung windows) show increased uptake (SUVmax 5,07 and SUVpeak 2,70). (**e**) Lung wedge resection reveals aspiration pneumonia with abscess formation, associated with colonies of Actinomyces (H&E, 40×). (**f**) Immunohistochemical staining for integrin α_v_β_6_ demonstrates positive alveolar and bronchial epithelium surrounding the inflamed area (40×). (**g**) Higher-power view demonstrates strong integrin α_v_β_6_ expression in the proliferating alveolar and bronchial epithelium surrounding the inflamed area (200×).

**Figure 5:**
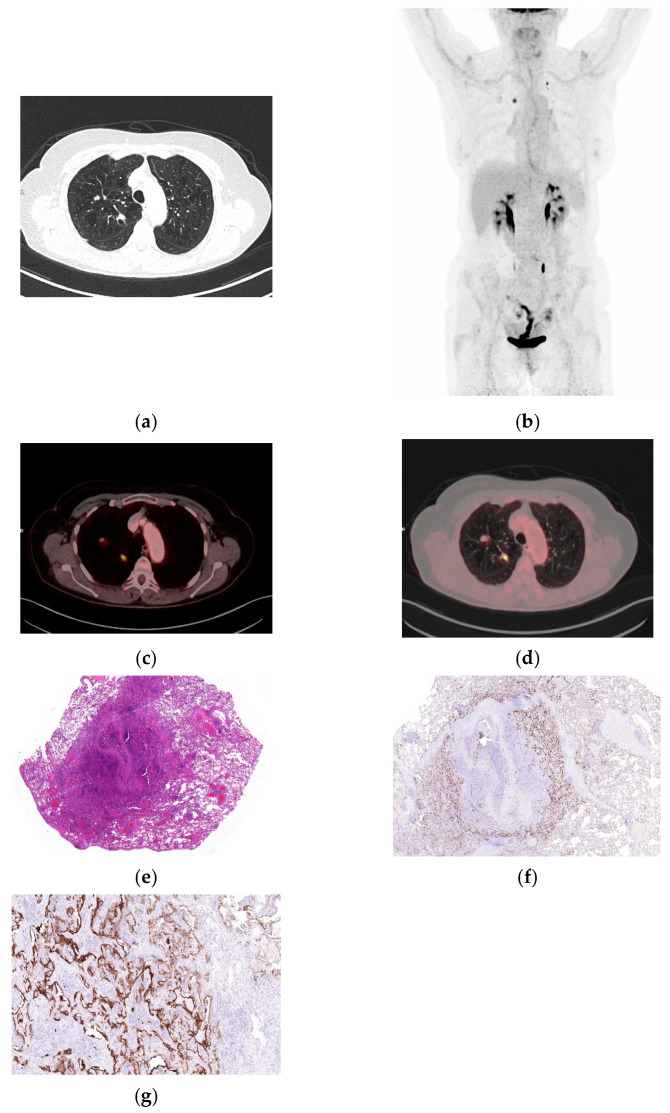
Chronic necrotizing pulmonary aspergillosis with increased FDG uptake. (**a**) Axial CT shows bilateral nodules. (**b**) Whole-body MIP shows FDG-avid lesions. (**c**,**d**) Fused axial ^18^FDG PET/CT images (soft tissue and lung windows) demonstrating increased uptake (SUVmax 9.18 and SUVpeak 3.10). (**e**) Lung wedge resection showing a circumscribed lesion with parenchymal necrosis containing Aspergillus hyphae, surrounded by lymphohistiocytic granulomatous inflammation (H&E, 40×). (**f**) Immunohistochemical staining shows integrin α_v_β_6_-positive alveolar and bronchial epithelial cells along the periphery of the inflamed and granulomatous area (40×). (**g**) Higher-power view demonstrates strong integrin α_v_β_6_ expression in the proliferating alveolar and bronchial epithelium along the periphery of the inflamed and granulomatous area (200×).

**Figure 6:**
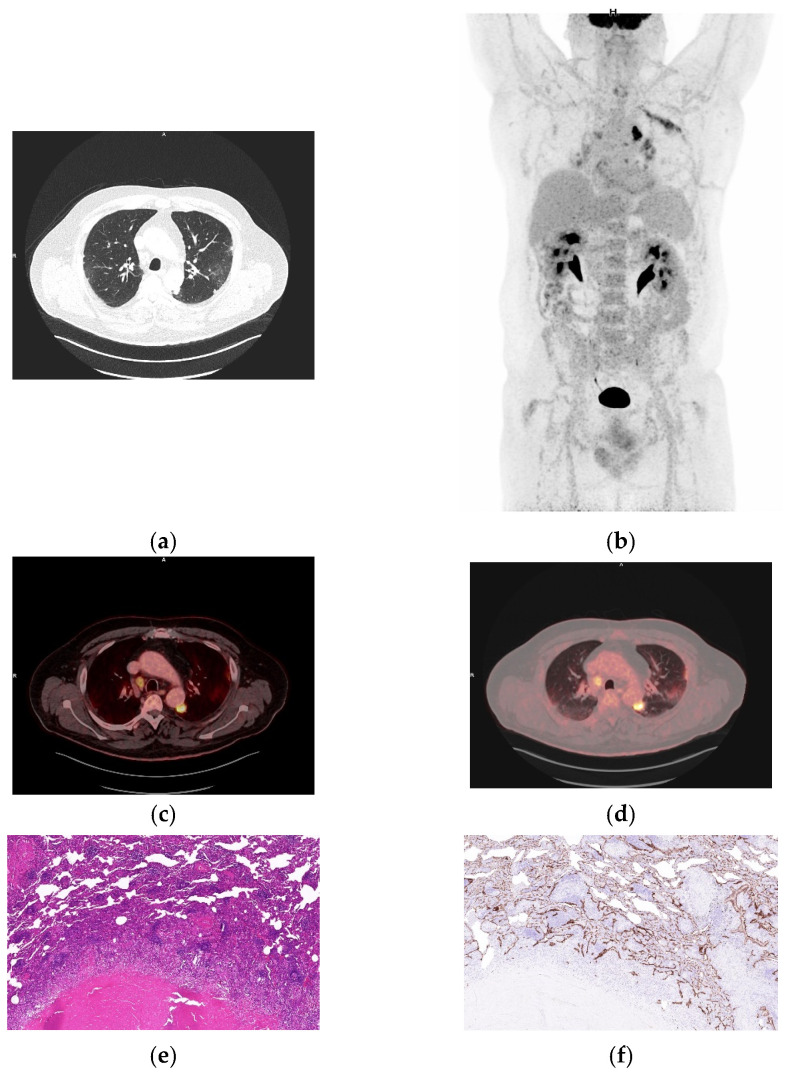
Granulomatosis with polyangiitis demonstrating intense FDG uptake. (**a**) Axial CT shows bilateral hilar and mediastinal lymphadenopathy. (**b**) Whole-body MIP demonstrating marked tracer uptake. (**c**,**d**) Fused axial ^18^FDG PET/CT images (soft tissue and lung windows) demonstrating a hypermetabolic lesion (SUVmax 18.07 and SUVpeak 9.90). (**e**) Lung tissue showing parenchymal necrosis bounded by lymphohistiocytic granulomatous inflammation with multinucleated giant cells (known case of Wegener’s granulomatosis) (H&E, 20×). (**f**) Immunohistochemical staining highlights a prominent proliferation of integrin α_v_β_6_-positive alveolar and bronchial epithelial cells surrounding areas with inflammatory and granulomatous reaction (40×).

**Figure 7:**
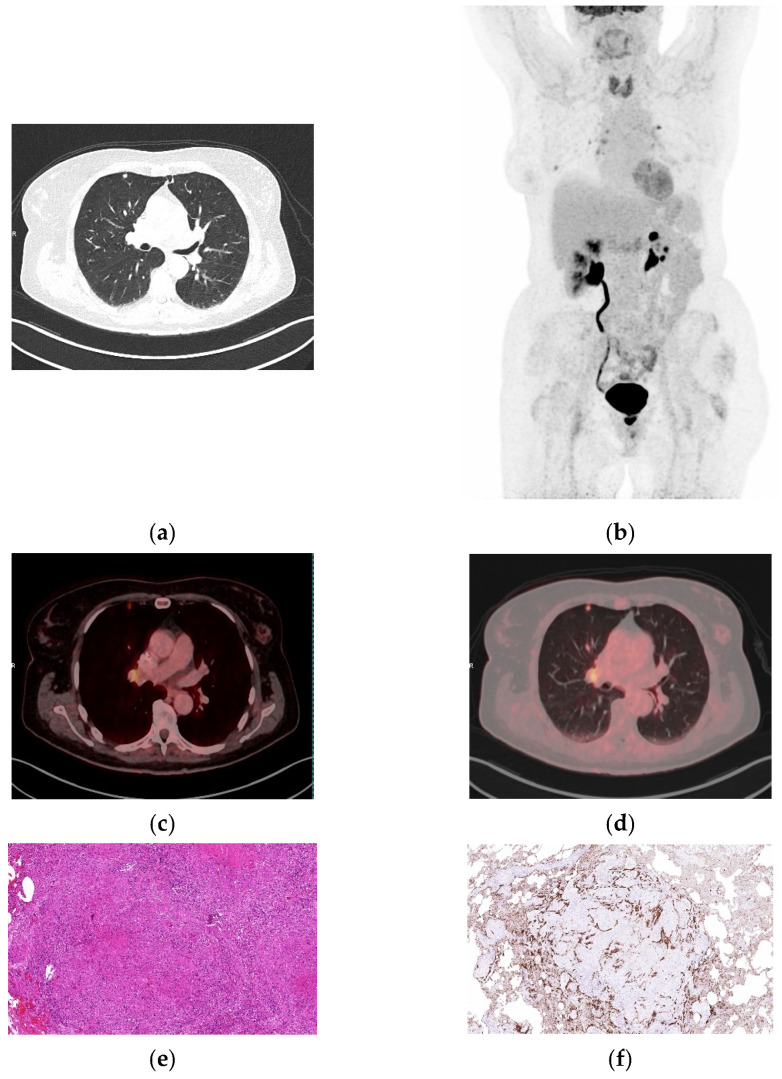
Necrotizing granulomatous inflammation with moderate FDG uptake. (**a**) Axial CT imaging demonstrated bilateral pulmonary lesions with bihilar lymphadenopathy. (**b**) Whole-body MIP shows FDG-positive lesions. (**c**,**d**) Fused axial ^18^FDG PET/CT images (soft tissue and lung windows) demonstrating bilateral FDG-positive pulmonary lesions (SUVmax 4.71 and SUVpeak 1.33). (**e**) Lung tissue showing granulomatous inflammation with multinucleated giant cells surrounding focal parenchymal necrosis (necrotizing sarcoid granulomatous) (H&E, 40×). (**f**) Immunohistochemical demonstrates a prominent proliferation of integrin α_v_β_6_-positive alveolar and bronchial epithelial cells distributed along the periphery of areas showing a granulomatous inflammatory reaction (40×).

Integrin α_v_β_6_ expression was detected in all 18 cases. Semi-quantitative integrated scoring showed strong overexpression in 16/18 lesions and moderate expression in 2/18, localized to the membrane of proliferating alveolar and bronchiolar epithelial cells surrounding areas of inflammation, necrosis, or fibrosis.

## 4. Discussion

Lung lesions are common imaging findings in clinical practice, with a variety of potential etiological agents. These lesions can pose a diagnostic dilemma as they can either represent malignancy or non-malignant mimickers that are potentially curable [16,17]. Resolving this dilemma prior to surgical excision is advantageous as the management and prognosis for malignancies is completely different compared to non-malignant lesions.

In this retrospective cohort of 18 pulmonary lesions that were surgically treated after being falsely classified as malignant on CT and PET/CT based on high tracer uptake, histopathology identified non-neoplastic inflammatory conditions in all cases. Immunohistochemical analysis revealed marked overexpression of integrin α_v_β_6_ in the proliferating alveolar and bronchial epithelium across these inflammatory lesions, with dense integrin-positive regions, which can cause high ^68^Ga-Trivehexin PET/CT uptake. These results show that integrin α_v_β_6_ upregulation is not specific to neoplastic processes but also occurs in the context of inflammation and tissue injury, limiting the ability of ^68^Ga-Trivehexin PET/CT to distinguish between malignant and inflammatory lesions when used alone for primary pulmonary lesion characterization [8,18].

Integrin α_v_β_6_ is a transmembrane adhesion receptor with minimal expression in healthy adult epithelia, but its expression is rapidly induced in proliferating epithelial cells undergoing injury, migration, and repair [4,19]. This injury-inducible profile has been well described in lung epithelium, where alveolar type II cells and bronchiolar epithelial cells upregulate integrin α_v_β_6_ in response to tissue damage or remodeling stimuli [20,21]. The biological function of integrin αvβ6 involves activation of latent transforming growth factor-β1 (TGF-β1), a key regulator of inflammation and fibrogenesis, linking epithelial repair programs to local immunoregulation and fibrotic pathways [21,22].

Literature comparisons show that integrin α_v_β_6_ IHC is positively expressed in about 54–100% of NSCLC cases with different intensities and a moderate-to-strong correlation between ^68^Ga-Trivehexin SUVmax and IHC scores (r = 0.631; *p* < 0.01) [8]. In another study conducted to assess the potential role of ^68^Ga-Trivehexin PET/CT in patients with head and neck squamous cell carcinomas and pancreatic ductal adenocarcinomas and their correlation with α_v_β_6_ integrin expression, it was found that integrin αvβ6 was highly expressed in lesions with higher uptake of ^68^Ga-Trivehexin, yet uptake was also reported around tumor sites [23]. These findings support our conclusion that integrin α_v_β_6_ upregulation, with strong membranous expression found in our inflammatory cases, which was limited to reactive proliferating alveolar/bronchiolar epithelium around necrotic or fibrotic foci, could reflect epithelial activation/repair rather than malignant transformation alone, while acknowledging that integrin α_v_β_6_ expression levels may be higher in many epithelial tumors.

The activation of TGF-β via α_v_β_6_ provides an explanation for the observed expression patterns in our inflammatory cases: epithelial injury and subsequent repair responses trigger integrin α_v_β_6_ expression as part of the regulatory milieu. Preclinical and clinical studies have documented that α_v_β_6_ expression increases not only in epithelial cancers but also in fibrotic lung disease, such as idiopathic pulmonary fibrosis (IPF), where elevated α_v_β_6_ levels correlate with disease severity [8,18]. Similarly, first-in-human integrin α_v_β_6_ PET tracers have demonstrated uptake in chronic lung fibrosis and post-injury epithelial remodeling, in addition to cancer, confirming that elevated tracer accumulation is associated with both processes [22].

The development of ^68^Ga-Trivehexin as a high-affinity PET ligand for integrin α_v_β_6_ has brought promise for epithelial cancer imaging because of its strong and selective binding to integrin α_v_β_6_, achieving high tumor-to-background contrast in several malignancies [10,24,25]. However, preliminary clinical experience has also revealed significant uptake in non-malignant injured or fibrotic lung tissue and other benign conditions, emphasizing that integrin α_v_β_6_ expression is not restricted to neoplasia [10,18,21]. Specifically, scans in patients with IPF have shown prominent radiotracer uptake in fibrotic areas, despite the absence of cancer [8,18]. These findings echo our data, demonstrating that integrin α_v_β_6_ PET tracers can generate false-positive signals in the setting of active inflammation and tissue repair, similar to the well-known limitations of ^18^F-FDG PET/CT in differentiating inflammation from malignancy.

Taken together, our histopathological, immunohistochemical, and imaging-related observations show that integrin α_v_β_6_ expression reflects epithelial activation rather than malignant transformation per se. Although integrin α_v_β_6_-targeted imaging may therefore be valuable for identifying areas of epithelial remodeling, it lacks specificity for epithelial neoplasia, as integrin α_v_β_6_ is also upregulated in inflammatory and reactive lesions [26,27]. This limitation must be carefully considered in clinical interpretation, particularly in pulmonary imaging, where inflammatory processes are common. Consequently, ^68^Ga-Trivehexin PET/CT is unlikely to reliably differentiate pulmonary non-small cell carcinoma—and probably epithelial neoplasms at other anatomical sites—from inflammatory or reactive lesions. Nevertheless, this imaging approach remains suitable for the detection of lymph node and soft-tissue carcinoma metastases [11,28,29,30].

The heterogeneous spectrum of pulmonary lesions mimicking malignancy warrants further investigation of the expression of integrin α_v_β_6_ in surgically resected specimens and the utility of 68Ga-Trivehexin PET/CT for preoperative differentiation. In the present study, integrin α_v_β_6_ was expressed by benign lesions confirmed by pathology, which were considered a surgical risk. Integrin α_v_β_6_ expression could therefore serve as a potential biomarker for these undetermined and non-malignant pulmonary lesions. Despite the known association of integrin β6 in lung cancer and its extrapulmonary malignancy counterparts, none of the integrin α_v_β_6_-positive lesions in the present study received a malignant diagnosis, suggesting the need to address integrin α_v_β_6_ expression in regard to early-stage lung cancers without other malignant lesions, where a comprehensive evaluation for malignancy remains ambiguous.

## 5. Conclusions

Our histopathological and immunohistochemical results emphasize that integrin α_v_β_6_ is upregulated not only in neoplastic lung tissue but also in inflammatory lesions undergoing epithelial repair and remodeling. Consequently, while ^68^Ga-Trivehexin PET/CT alone cannot reliably differentiate neoplastic from inflammatory primary pulmonary lesions, as both demonstrate significant tracer uptake, it carries practical significance in enhancing the staging of lymph node and brain metastases in confirmed NSCLC, along with possible uses in observing fibrotic lung diseases. This parallels the diagnostic challenge of ^18^F-FDG PET/CT false positives in inflammatory conditions and highlights the need for cautious interpretation and histopathological confirmation when integrin αvβ6 PET imaging indicates high integrin expression.

Based on the observed integrin expression in inflammatory, non-neoplastic lesions, the use of integrin-targeted antibody–drug conjugates or radiolabeled compounds for cancer therapy should be considered only after histopathological confirmation of malignancy in the targeted lesion.

## Data Availability

The original contributions presented in this study are included in the article. Further inquiries can be directed to the corresponding author.
